# Influence of breast compression pressure on the performance of population-based mammography screening

**DOI:** 10.1186/s13058-017-0917-3

**Published:** 2017-11-28

**Authors:** Katharina Holland, Ioannis Sechopoulos, Ritse M. Mann, Gerard J. den Heeten, Carla H. van Gils, Nico Karssemeijer

**Affiliations:** 1 Department of Radiology and Nuclear Medicine, Radboud University Medical Center, PO Box 9101, 6500 HB Nijmegen, The Netherlands; 2 Department of Radiology/Biomedical Engineering and Physics, Academic Medical Center Amsterdam, PO Box 22660, 1100 DD Amsterdam, The Netherlands; 3 Julius Center for Health Sciences and Primary Care, University Medical Center Utrecht, PO Box 85500, 3508 GA Utrecht, The Netherlands

**Keywords:** Mammography, Breast compression, Breast cancer screening, Performance measures

## Abstract

**Background:**

In mammography, breast compression is applied to reduce the thickness of the breast. While it is widely accepted that firm breast compression is needed to ensure acceptable image quality, guidelines remain vague about how much compression should be applied during mammogram acquisition. A quantitative parameter indicating the desirable amount of compression is not available. Consequently, little is known about the relationship between the amount of breast compression and breast cancer detectability. The purpose of this study is to determine the effect of breast compression pressure in mammography on breast cancer screening outcomes.

**Methods:**

We used digital image analysis methods to determine breast volume, percent dense volume, and pressure from 132,776 examinations of 57,179 women participating in the Dutch population-based biennial breast cancer screening program. Pressure was estimated by dividing the compression force by the area of the contact surface between breast and compression paddle. The data was subdivided into quintiles of pressure and the number of screen-detected cancers, interval cancers, false positives, and true negatives were determined for each group. Generalized estimating equations were used to account for correlation between examinations of the same woman and for the effect of breast density and volume when estimating sensitivity, specificity, and other performance measures. Sensitivity was computed using interval cancers occurring between two screening rounds and using interval cancers within 12 months after screening. Pair-wise testing for significant differences was performed.

**Results:**

Percent dense volume increased with increasing pressure, while breast volume decreased. Sensitivity in quintiles with increasing pressure was 82.0%, 77.1%, 79.8%, 71.1%, and 70.8%. Sensitivity based on interval cancers within 12 months was significantly lower in the highest pressure quintile compared to the third (84.3% vs 93.9%, *p* = 0.034). Specificity was lower in the lowest pressure quintile (98.0%) compared to the second, third, and fourth group (98.5%, *p* < 0.005). Specificity of the fifth quintile was 98.4%.

**Conclusion:**

Results suggest that if too much pressure is applied during mammography this may reduce sensitivity. In contrast, if pressure is low this may decrease specificity.

## Background

In mammography, breast compression is applied to reduce the thickness of the breast. This results in improved image quality because tissue superposition and x-ray scatter are reduced, while it limits the required dose [1–4]. In addition, with a compression paddle the breast can be kept in a fixed position, which reduces the risk of motion artefacts and image blurring.

Mammography devices measure and display compression force during the imaging procedure. However, there are no quantitative guidelines regarding the compression force a radiographer should apply for acquisition of an adequate mammogram. In practice, compression force in mammography varies widely among radiographers, screening centers, and countries [5–10]. A disadvantage of compression is that many women complain about discomfort and pain which might influence their participation in screening [11–13]. A reduction in compression force has therefore been suggested to encourage screening attendance [14].

Although mammography systems display the compression force applied by the compression paddle to the breast, it is the pressure, which is defined as the compression force divided by the contact area between breast and compression paddle, that determines how much the tissue is compressed. It is therefore likely that the pain experienced by women undergoing mammography is more related to pressure than to force. The same force applied to a small or a large breast leads to different pressures. Pressure depends on the force and the contact area between the breast and the paddle, which depends on the breast size and the deformation and shape changes of the breast during compression. In case of a large contact area, the force is distributed over a larger area, leading to a lower pressure (force per unit area) compared to a small area. In a study by de Groot et al. [15] the force-standardized compression protocol was replaced by a pressure-standardized protocol using a recently developed paddle [16]. It was found that pain was reduced with pressure standardization, while average glandular dose remained unchanged.

While it is widely accepted that firm breast compression is needed to ensure acceptable image quality, guidelines remain vague about how much compression should be applied. For example, the European guideline states that "the breast should be properly compressed, but no more than is necessary to achieve a good image quality" [17]. A quantitative parameter indicating the amount of compression is not presented. Consequently, little is known about the relationship between the amount of breast compression and breast cancer detectability. Furthermore, it has been reported that too much compression, as applied during spot compression, can lead to dissolving of suspicious densities in some cases [18–20]. Therefore, this retrospective study aims to investigate if the level of breast compression can impact screening performance, using pressure to characterize the level of compression.

## Methods

### Screening data

In this retrospective study, mammograms that were acquired in the Dutch breast cancer screening program are used. In this population-based program, women between 50 and 75 years of age are invited for a screening examination every 2 years. A consecutive series of mammograms acquired in one screening unit were collected. Raw mammograms acquired in this unit were archived between 2003–2012, except for a 4-month period in 2009 due to a technical issue. The images of the 135,640 available examinations were acquired on Lorad Selenia systems (Hologic, Inc., Danbury, CT, USA). The majority of the images were acquired with a flexible paddle. Cancer status information was obtained from the screening registration system and the Netherlands Cancer Registry. Written informed consent was not required for this study as women automatically consent to the use of their anonymized data for scientific purposes by participating in screening unless they object. Data of participants who objected to the use of their data were removed. A waiver to use the anonymized mammograms for research was obtained from the research ethics committee of the Radboud University Medical Center.

In this screening program, medio-lateral oblique (MLO) and cranio-caudal (CC) view images were always acquired at the first screening. However, during the study period, screening guidelines recommended that during subsequent rounds CC images be acquired in case of severe density, post-surgical changes, and if the technician suspected that this would be helpful in the clarification of an unclear structure. Therefore, CC view images are available only in about 60% of the examinations. There are systematic differences between MLO and CC views in force and pressure. Therefore, to exclude these differences and to prevent a bias towards abnormal and dense breasts we used only MLO view images in this study.

Screen-detected cancers or true positives (TPs) are defined as the cancers diagnosed after a recall of a woman for additional diagnostic tests. Interval cancers or false negative (FN) examinations are defined as cancers that were diagnosed within 24 months after a screening examination that did not lead to a recall (negative screening mammogram) and before attending the next scheduled screening examination. False positive (FP) examinations are exams of women recalled for additional tests in which no breast cancer was diagnosed, and true negatives (TNs) are examinations that did not lead to a recall and after which no breast cancer was diagnosed within 24 months before the attendance to another screening round.

### Image analysis

Compression pressure was determined retrospectively using a research version of the software Volpara (v1.5.0, Volpara Health Technologies, Wellington, New Zealand). The algorithm determines the contact area between the breast and the compression paddle by image analysis as described in [21]. The contact area measured with Volpara has been validated against manual segmentations from video images and a correlation of *R* = 0.978 (confidence interval (CI) 0.972–0.982) was found [22]. Pressure is computed by taking the compression force measurement from the imaging device, which is stored in the image header, and dividing it by the estimated contact area. Volpara software was also used to determine breast volume and dense tissue volume, which were used to compute percent dense volume. For the TN examinations and bilateral findings, the values obtained for the left and right MLO views were averaged. For all other examinations the values for the side with the finding or cancer was used.

### Data analysis

The relationships between breast volume and force and pressure were investigated using heatmaps. The pressure measurements were used to divide the dataset into quintiles of increasing pressure. Within the five groups the following performance measures were determined: recall rate (number of true positive and false positive findings per 1000 women screened), false positive rate (number of false positive findings per 1000 women screened), screen-detected breast cancer rate (number of screen-detected breast cancers per 1000 women examined), specificity (number of true negative findings divided by the number of exams without cancer diagnosis; TN/(TN + FP)), and positive predictive value (number of screen-detected cancers divided by the number of recalls; TP/(TP + FP)) as performed in a previous publication [23]. Additionally, the interval cancer rate (number of interval cancers per 1000 examinations), the program sensitivity (number of screen-detected cancers divided by the sum of screen-detected cancers and interval cancers diagnosed before the next screening round), and 12-month sensitivity (program sensitivity using only the interval cancers diagnosed within 12 months after examination) were determined. The 12-month sensitivity may be used to translate results to the context of an annual screening program.

Many women in the dataset had more than one screening examination. To account for correlation between examinations of the same woman, we used generalized estimating equations (GEE) using the ‘independence’ correlation structure. As it is known that the sensitivity of mammography is lower in women with higher breast density [24–26], breast volume and percent dense volume were included in the models to adjust for their potentially confounding effect. Breast volume and percent dense volume were transformed using the natural logarithm to obtain data which approximated normal distributions. Pair-wise testing was applied to assess differences between the groups on the sensitivity and specificity measurements. Correction for multiple testing was applied using the Tukey method and a *p* value below 0.05 was considered significant. GEE was used for each performance measure separately.

For each pressure group, the distribution of the different cancer types and their detection at screening or as interval cancers, using the non-corrected data, was also investigated. For this, the following categories were used: ductal carcinoma in situ (DCIS), invasive ductal carcinoma (IDC), invasive lobular carcinoma (ILC), ‘other’, and unknown.

Statistical analysis was performed using R version 3.3.1.

## Results

In total 135,640 examinations were available. Excluding examinations with unknown screening outcome (*n* = 72), examinations without a percent dense volume, contact area or force measurement available (*n* = 2,673), and interval cancers diagnosed more than 24 months after the examination (*n* = 119), a total of 132,776 examinations of 57,179 women were included in the analysis. The mean age at the time of the examination was 59.2 years, and 39,449 women contributed two or more examinations to the dataset while 17,730 women contributed one examination (not necessarily their first screening mammogram).

To stratify the examinations into five groups of equal size, thresholds on the pressure estimates were applied at 7.7 kPa, 9.3 kPa, 10.8 kPa, and 13.0 kPa. When a pressure measurement equals the threshold, the lower class is assigned to the examination. The mean breast volumes, percent dense volumes, forces, and pressures of the five groups are listed in Table 1. As expected, it can be seen that increasing compression pressure correlated with decreasing breast volume and increasing breast density and force.

**Table 1:** Number of screening examinations and mean measures

	Total	Group 1 ≤ 7.7 kPa	Group 2 7.7–9.3 kPa	Group 3 9.3–10.8 kPa	Group 4 10.8–13.0 kPa	Group 5 > 13.0 kPa
Number of examinations	132,776	26,490	26,617	26,539	26,549	26,581
Mean breast volume (cm^3^)	974	1511	1135	928	755	540
Mean percent dense volume (%)	7.8	5.7	6.6	7.5	8.4	10.7
Mean force (N)	125.9	112.6	121.5	125.9	130.7	138.9
Mean pressure (kPa)	10.5	6.6	8.5	10.0	11.8	15.6

Thresholds to form the groups were applied at 7.7 kPa, 9.3 kPa, 10.8 kPa, and 13 kPa

An examination with a pressure measurement equal to the threshold was assigned to the lower group

**Fig. 1:**
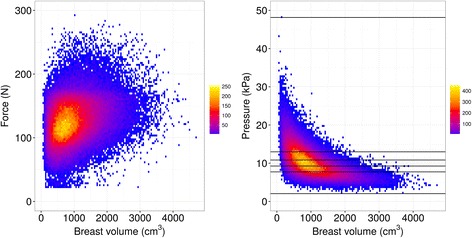
Measurements of force and pressure in relation to the breast volume. The color code represents the number of examinations in each bin. The *horizontal lines* in the *right* panel indicate the thresholds used to obtain the five pressure groups and the minimum and maximum pressure value observed

Heatmaps showing the variations in force and pressure with breast volume are shown in Fig. 1 illustrating the difference between force and pressure. To indicate the pressure groups, horizontal lines were added to the pressure distribution according to the thresholds applied to form the quintiles. It is observed that breasts of the same size are imaged using a wide range of forces. At the same time a trend is indicating that larger breasts are imaged with higher forces, so some adjustment to the individual breast takes place. The pressure distribution shows, however, that the very large breasts are imaged with a low pressure and therefore most of these cases are in the first pressure category. Additionally, it is observed that the first pressure group contains the entire range of breast sizes, while extremely high compression is mainly a problem for small breasts. This is due to medium and high forces, as shown in the left heatmap, being distributed over a small contact area, leading to high pressure.

Table 2 gives an overview of the screening outcomes for the complete cohort stratified by the five compression pressure groups. Screening performance measures are displayed in Table 3. Results suggest that, at high compression pressure, sensitivity is reduced (82.7%, 77.4%, 79.7%, 70.0%, and 68.2% for the five groups, respectively).

**Table 2:** Number of screen-detected cancers, interval cancers, false positive examinations and true negative examinations

		Group 1		Group 2		Group 3		Group 4		Group 5	
	Total	*n*	%	*n*	%	*n*	%	*n*	%	*n*	%
Total examinations	132,776	26,490	20.0	26,617	20.0	26,539	20.0	26,549	20.0	26,581	20.0
Screen detected cancers	833	177	21.2	164	19.7	188	22.6	152	18.2	152	18.2
Interval cancers	269	37	13.8	48	17.8	48	17.8	65	24.2	71	26.4
Interval cancers detected within 12 months after the examination	110	19	17.3	15	13.6	13	11.8	26	23.6	37	33.6
False positive examinations	2192	486	22.2	381	17.4	404	18.4	426	19.4	495	22.6
True negative examinations	129,482	25,790	19.9	26,024	20.1	25,899	20.0	25,906	20.0	25,863	20.0

The 12-month interval cancers are included in the interval cancers

**Table 3:** Unadjusted screening performance measurements

	Total	Group 1	Group 2	Group 3	Group 4	Group 5
Recall/1000	22.8	25.0	20.5	22.3	21.8	24.3
False positives/1000	16.5	18.4	14.3	15.2	16.1	18.6
Screen-detected cancers/1000	6.3	6.7	6.2	7.1	5.7	5.7
Interval cancers/1000	2.0	1.4	1.8	1.8	2.5	2.7
Program sensitivity (%)	75.6	82.7	77.4	79.7	70.0	68.2
12-month sensitivity (%)	88.3	90.3	91.6	93.5	85.4	80.4
Specificity (%)	98.3	98.2	98.6	98.5	98.4	98.1
Positive predictive value (%)	27.5	26.7	30.1	31.8	26.3	23.5

The sensitivity is calculated with all interval cancers, the 12-month sensitivity is calculated with the interval cancers that were detected within 12 months after the examination

**Fig. 2:**
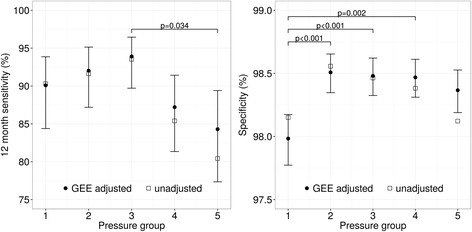
Measurements of 12-month sensitivity and specificity. Measurements of 12-month sensitivity and specificity of the five pressure groups of unadjusted data (*squares*) and after adjustment with generalized estimating equations (*GEE*) for multiple screening rounds, breast volume, and percent dense volume including 95% CI (*circles*). Statistically significant differences between pairs of groups of the GEE adjusted data are indicated

Results from the GEE models are shown in Table 4, confirming the decrease in sensitivity at high pressure observed in the unadjusted data. The sensitivity of the five pressure groups is 82.0%, 77.1%, 79.8%, 71.1%, and 70.8%, respectively, while the 12-month sensitivity is 90.1%, 92.0%, 93.9%, 87.2%, and 84.3%, respectively. There is a statistically significant difference in the 12-month sensitivity between the third and the fifth group (*p* = 0.034). Even though this is the only significant difference between groups on the sensitivity measurements, a considerable difference can be observed between the first three pressure groups and the last two pressure groups. Results also show a trend that women with mammograms in the lowest pressure group are recalled more often. This leads to a higher false positive rate, and lower specificity and positive predictive value. The specificity was found to be significantly lower in the first group (98.0%) compared to the second (*p* < 0.001), third (*p* < 0.001), and fourth (*p* = 0.002) group which all have a specificity of 98.5%. The 12-month sensitivity and the specificity are displayed in Fig. 2 with and without correction for confounders.

**Table 4:** Adjusted screening performance measurements with 95% confidence intervals using GEE to account for correlations between mammograms of the same woman and to correct for the confounders percent dense volume and breast volume

	Group 1	Group 2	Group 3	Group 4	Group 5
Recall/1000	26.1 (24.0–28.5)	20.8 (19.1–22.6)	22.0 (20.3–23.9)	21.0 (19.3–22.8)	22.2 (20.3–24.3)
False positives/1000	20.0 (18.1–22.1)	14.8 (13.4–16.4)	15.1 (13.7–16.6)	15.2 (13.8–16.8)	16.2 (14.6–18.0)
Screen-detected cancers/1000	6.1 (5.2–7.2)	5.9 (5.0–6.9)	6.9 (6.0–8.0)	5.7 (4.9–6.7)	5.9 (4.9–7.0)
Interval cancers/1000	1.3 (0.9–1.8)	1.6 (1.2–2.2)	1.6 (1.2–2.1)	2.2 (1.7–2.8)	2.3 (1.7–3.0)
Program sensitivity (%)*	82.0 (75.6–87.0)	77.1 (70.9–82.4)	79.8 (74.2–84.4)	71.1 (64.5–79.8)	70.8 (63.6–77.1)
12 month sensitivity (%)**	90.1 (84.4–93.9)	92.0 (87.2–95.1)	93.9^a^ (89.7–96.5)	87.2 (81.3–91.4)	84.3^a^ (77.3–89.4)
Specificity (%)	98.0^a,b,c^ (97.8–98.2)	98.5^a^ (98.3–98.7)	98.5^b^ (98.3–98.6)	98.5^c^ (98.3–98.6)	98.4 (98.2–98.5)
Positive predictive value (%)	23.5 (20.2–27.3)	28.7 (25.0–32.7)	31.5 (27.9–35.3)	27.3 (23.7–31.1)	26.7 (22.9–30.8)

Pair-wise testing was applied on the sensitivity and specificity measurements and each subscript letter denotes a pair of pressure categories whose measurements differ significantly from each other

The Tukey method was used for correction for multiple testing and a *p* value below 0.05 was considered significant

The programm sensitivity (*) is calculated with all interval cancers, the 12-month sensitivity (**) is calculated with the interval cancers that were detected within 12 months after the examination

The investigation of the distribution of DCIS, IDC, ILC, and the remaining other types of cancers for the different pressure categories shows that only a few DCIS cases are found among the interval cancers. The proportion of lobular and other types of cancers is relatively high for the interval cancers in the highest pressure group compared to the other pressure groups and the proportion of lobular and other types of cancers for the screen-detected cancers (data not shown). Because of the low number of cancers in each subgroup, statistical analysis of subgroup differences was not performed.

## Discussion

Given the reasons for using mechanical breast compression during mammographic imaging (reduction of tissue superposition, scatter, dose, and possibility of motion among other image quality-related benefits), it was expected that screening outcomes would be negatively impacted if the compression pressure applied was too low, as found in our results. As could have been foreseen, applying insufficient compression lowers the specificity of mammography, perhaps due to a lack of minimization of tissue superposition. In this context, it should be highlighted that the overall compression force, and therefore pressure, is rather high in the Netherlands compared to other countries [5, 6, 8]. Hence, the loss of performance due to insufficient compression may be a more common issue in general than that found in this study. The finding demonstrates that an adequate level of compression is necessary to obtain good image quality and achieve a low recall rate, and stresses the need for techniques to apply compression at the right level. Although one should be careful with extending our conclusions to other populations, since screening policies vary from country to country (e.g., recall rates are higher in the US than in Europe), we note that our study sample is representative for breast cancer screening in Europe where low recall rates, as in our study, are common [27].

Although some reports were published about reduced visibility of a subset of tumors in spot compression [18–20], which typically are made with stronger compression, it was not a priori expected that high compression levels in screening mammography would have a large negative impact on screening outcomes. However, it seems that applying a higher than needed compression actually has a stronger negative effect on lesion visibility than applying insufficient compression, even when correcting for the confounding effects of breast density and volume, resulting in a lower sensitivity. Even though the difference in sensitivity did not reach statistical significance when corrected for confounding factors, except for the 12-month sensitivity between group 3 and group 5, the reduction was considerably larger than that suffered due to applying low compression.

It is not straightforward to identify the underlying cause for reduced sensitivity at high compression levels. This reduction in sensitivity could be due to either malignancies not being seen or being seen but mischaracterized, or due to both types of errors. Reduced visibility of certain tumors under high compression might be due to their composition. It can be reasoned that softer tumors may become less conspicuous with high compression because the cancer tissue may spread out and lose contrast. Another reason could be that lesion types that are detected because of architectural changes of the breast parenchyma are less conspicuous under lower and higher pressure. This is supported by the distribution of ILC over the five groups. However, it is hard to say with the given data whether a specific type of cancer is more often missed because of too low or too high pressure. The different cancer types should be kept in mind when investigating the relationship between pressure and screening performance in future studies, with other, perhaps larger, datasets. In terms of mischaracterization, vascularization might play a role. Since invasive cancers are often highly vascularized, strong compression may lead to a reduction in blood flow [28, 29], leading to both a decrease in contrast and a reduction in the perceived suspiciousness of the finding, causing misinterpretation of its probability of malignancy [30].

The force and the pressure distributions were displayed in relation to the breast volume. The first pressure group contains the entire range of breast volumes. For women with small breasts, the low pressure is caused by a low force. For larger breasts, the contact area is larger so that even a medium or high force leads to a low pressure. An extremely high pressure is only observed for small breasts and is caused by too much force. Therefore, a compression recommendation based on force cannot solve the over-compression of small breasts and the under-compression of large breasts, as the force measure is independent of the individual breast characteristics. On the other hand, a pressure-guided compression could prevent extreme compressions of small breasts and too low compressions of larger breasts, as the pressure measurement depends on the breast size, shape, and stiffness.

The relationship between compression pressure and screening performance measures was also investigated in the recent study of Moshina et al. [31] with data from the Norwegian breast cancer screening program. About 260,000 examinations were categorized into low, medium, and high pressure groups, and the performance measures were calculated using GEE including a correction for fibroglandular tissue volume and age. Also in the Norwegian data, a decrease in sensitivity was observed with increasing pressure which supports the main finding of our study. However, their finding that low compression pressure is associated with more favorable performance measures was not confirmed in our study. We observed a lower specificity for examinations in the lowest pressure group. It should, however, be noted that the results of both studies are not directly comparable. Different confounding factors were considered in the Norwegian study (fibroglandular tissue volume and age) than in this study (percent dense volume and breast volume), in combination with different groupings (tertiles vs quintiles).

In this study, we computed the pressure to the entire breast as a single value, based on the overall force and contact area. Because we used MLO views, the pectoral muscle is also included in the contact area. Therefore, the computed pressure might not accurately reflect the pressure on the breast tissue in regions where most lesions are located. Since Dustler et al. [32] found that the resulting pressure distribution during breast compression is not uniform, and that in some cases the pressure is highest in the pectoral muscle, this is a limitation of our study. On the other hand, without knowing the mechanism that might lead to reduced sensitivity at high pressure, the issue of pressure measurements remains open. For instance, if the lesions were missed due to a reduced blood flow into the breast, perhaps the pressure at the posterior border of the breast might be an important factor.

Another limitation of this study is that CC views, and the per-view lesion sensitivity and specificity, were not included due to the fact that CC views were not acquired in a substantial portion of the screening examinations at the time of data acquisition. In essence, this means that the presented results are based on the pressure applied for the acquisition of the MLO view only, while the screening outcomes are calculated based on the whole examination, which in the majority of examinations also included CC views. To investigate the effect of compression in CC view images on the screening performance, a dataset for which both views are available should be used. Finally, although this is a dataset spanning many years and therefore includes examinations in which various radiographers performed the acquisitions and various radiologists interpreted them, it is still a single-site study with all images acquired with a single mammographic system model. It is not expected, however, that compressions performed with other systems should have an impact on breast compression pressure and its relationship to screening outcomes.

Our retrospective analysis is performed with mammograms acquired in screening practice. Although the results and conclusions are based on a large sample of cases, evidence for our findings would become stronger if mammograms of individual women could be obtained who were repeatedly imaged with different compressions. Since such evidence is currently lacking, we feel that it would not be appropriate to recommend an optimal pressure range based on this study alone. Further studies are needed to confirm our findings, ideally including a study with repeated imaging at different predefined pressures, to investigate lesion visibility as function of pressure.

## Conclusion

In conclusion, this study shows a relation between the applied pressure and the performance of screening mammography even when taking into account confounding effects. The recall rate, false positive rate, and specificity were affected negatively in the compression category with the lowest pressure, while the sensitivity was reduced in the categories with high pressure. Since this is the first time this has been reported, more research to confirm potential effects of pressure on screening performance measures is necessary because more attention to a meaningful standardization of compression levels might improve mammography in the future.

## Abbreviations

CC: Cranio-caudal
DCIS: Ductal carcinoma in situ
FN: False negative
FP: False positive
GEE: Generalized estimating equations
IDC: Invasive ductal carcinoma
ILC: Invasive lobular carcinoma
MLO: Medio-lateral oblique
TN: True negative
TP: True positive
